# Antioxidant and Glycemic Regulatory Properties Potential of Different Maturity Stages of Leaf of Ceylon Cinnamon (*Cinnamomum zeylanicum* Blume)* In Vitro*

**DOI:** 10.1155/2019/2693795

**Published:** 2019-07-18

**Authors:** Walimuni Prabhashini Kaushalya Mendis Abeysekera, Sirimal Premakumara Galbada Arachchige, Walimuni Kanchana Subhashini Mendis Abeysekera, Wanigasekera Daya Ratnasooriya, Hela Medawattegedara Upeksha Indeewari Medawatta

**Affiliations:** ^1^Herbal Technology Section, Industrial Technology Institute (ITI), 503A Halbarawa Gardens, Malabe, Sri Lanka; ^2^Department of Sciences and Social Sciences for Nursing, Faculty of Nursing, University of Colombo, Sri Lanka; ^3^Department of Agricultural Technology, Faculty of Technology, University of Colombo, Sri Lanka; ^4^Department of Zoology, Faculty of Science, University of Colombo, Colombo 3, Sri Lanka

## Abstract

Dichloromethane:methanol (1:1, v/v) extracts of different maturity stages (immature, partly mature, and mature) of authenticated leaves of Ceylon cinnamon (CC) were used in this study. Antioxidant properties [total polyphenolic content (TPC) and total flavonoid content (TFC), 1, 1-diphenyl-2-picryl-hydrazyl (DPPH), 2-azino-bis (3-ethylbenzothiazoline-6-sulfonic acid (ABTS)), oxygen radical absorbance capacity (ORAC), and ferric reducing antioxidant power (FRAP)] and glycemic regulatory properties [antiamylase (AA); antiglucosidase (AG)] were evaluated using 96-well microplate based bio assays* in vitro* (TPC, TFC, DPPH, ABTS, ORAC n=4 each; FRAP, AA, AG n=3 each). Results clearly revealed significant differences (p<0.05) among different maturity stages of leaf of CC for both antioxidant and glycemic regulatory properties (except AG activity). The mean antioxidant and glycemic regulatory activities of immature, partly mature, and mature leaves ranged from TPC: 0.68 ± 0.02–22.35 ± 0.21 mg gallic acid equivalents/g of sample (GS); TFC: 0.85 ± 0.01–4.68 ± 0.06 mg quercetin equivalents/GS; DPPH: 0.42 ± 0.01–27.09 ± 0.65 mg Trolox equivalents (TE)/GS; ABTS: 3.57 ± 0.10–43.91 ± 1.46 TE/GS; ORAC: 0.71 ± 0.01–18.70 ± 0.26 TE/G, FRAP: 0.31 ± 0.02–69.16 ± 0.52 TE/GS; and AA: 18.05 ± 0.24–36.62 ± 4.00% inhibition at 2.5 mg/mL. Mature leaf had the highest antioxidant and AA activities for all the assays investigated. In contrast, immature leaf had the lowest. The order of potency for antioxidant and AA activities was mature leaf > partly mature leaf > immature leaf. This is the first study to report on antioxidant and glycemic regulatory properties of different maturity stages of leaf of Ceylon cinnamon and highlights its potential use in management of oxidative stress-associated chronic diseases including diabetes mellitus.

## 1. Introduction

Free radicals, reactive oxygen, and nitrogen species in human body are derived from either various metabolic processes or to exposure of different physiochemical conditions [1]. Both enzymatic and nonenzymatic antioxidant defense mechanisms play a vital role to counterbalance the effect of free radicals/oxidants. However, imbalanced antioxidant defense mechanisms ensure a condition known as oxidative stress which is closely related and tightly linked with the pathophysiological processes of numerous degenerative diseases including diabetes mellitus [2].

Diabetes mellitus is a chronic metabolic disease characterized by hyperglycemia due to the defects in insulin secretion, action, or both and it is increasing at an epidemic proportion throughout the world [3, 4]. Recent statistics reported that there were 425 million people with diabetes worldwide in year 2017 and this number is predicted to be 629 million people in year 2045 [5]. Although current antidiabetic drugs and insulin regimes are very effective in managing diabetes mellitus still there is no permanent cure for this disease [6]. Therefore, search of novel drug leads/functional foods from natural sources preferably from medicinal plants with no/minimum side effects is timely important. As such alpha amylase and alpha glucosidase inhibitors are key targets since these two enzymes play a key role in digestion of carbohydrates [7] and several research findings too highlighted that antioxidants such as polyphenolics involve in inhibition of alpha amylase and alpha glucosidase enzymes [8–11]. Further, many studies demonstrated that oxidative stress plays a crucial role in associated pathological processes of diabetes mellitus [12–16] and has it been identified as the root cause underlying the development of insulin resistance, *β*-cell dysfunction, and impaired glucose tolerance in type 2 diabetes mellitus (T2DM). Perhaps, management of oxidative stress via antioxidant therapy has shown beneficial effects in management of oxidative stress-associated pathologies in diabetes patients [17].

Cinnamon is one of the earliest known and most popular spices used by mankind. It is the dried inner bark of several tree species of the genus* Cinnamomum *[18, 19]. Historically cinnamon was among the most expensive commodities traded world over [18, 19] and at present it is the fifth most expensive spice in the world [20]. Currently cinnamon obtained from four* Cinnamomum *species such as* C. zeylanicum *Blume (*C. verum *Presl) (Ceylon cinnamon/True cinnamon),* C. aromaticum *Presl (*C. *cassia/Chinese cinnamon/Chinese cassia),* C. burmannii *(Indonesian cinnamon/Indonesian cassia), and* C. loureiroi *(Vietnamese cinnamon/Vietnamese cassia/Saigon cinnamon) dominate the cinnamon trade worldwide [21]. Among them Ceylon cinnamon (*C. zeylanicum *Blume) is the true cinnamon where all the other cinnamons (Chinese cinnamon, Indonesian cinnamon, and Vietnamese cinnamon) are cassia types [18]. True cinnamon has unique characteristics in terms of processing of cinnamon bark, distinctive taste, and aroma [22] and also contains least amount of coumarin, the carcinogenic compound [23]. Further, it contributes nearly 10% of total cinnamon exports worldwide and which accounts nearly 32.9% of total export earnings of cinnamon worldwide [20]. Although the main application of Ceylon cinnamon as a spice and a flavoring agent it is also used in traditional medical system of Sri Lanka and in folklore to treat variety of disorders [24–27]. Further, this traditional knowledge of use of Ceylon cinnamon in medicine has been scientifically validated and reported to have several biological activities world over. The reported biological activities of Ceylon cinnamon were particularly on its bark and reported health benefits of leaf of Ceylon cinnamon is extremely rare [20, 28, 29]. We have previously reported leaf of Ceylon cinnamon as a rich source of natural antioxidants and possess antioxidant and antidiabetic activities through multiple mechanisms [29]. However, variation of antioxidants and antioxidant and antidiabetic activities of different maturity stages of leaf of Ceylon cinnamon is not reported to date. The present study therefore evaluates the antioxidant and glycemic regulatory properties potential of different maturity stages of leaf of Ceylon cinnamon* in vitro.*

## 2. Materials and Methods

### 2.1. Materials

#### 2.1.1. Collection of Leaf Samples of Ceylon Cinnamon

Different maturity stages (immature, partly mature, and mature) of fresh cinnamon leaves were collected from cinnamon cultivations of L.B spices (Pvt) Ltd., Aluthwala, Galle, Sri Lanka. Leaf samples were authenticated (voucher number CZB-KA) by Mr. N.P.T. Gunawardena, Officer In-Charge, National Herbarium, Department of National Botanic Gardens, Peradeniya, Sri Lanka. The specimens of leaf samples (HTS-CIN-1) and photographic evidence were deposited at the Pharmacognosy Laboratory, Herbal Technology Section, Industrial Technology Institute, Sri Lanka. Fresh leaves were oven dried at 45°C for overnight and powdered. Powdered leaf samples were stored at −20°C until use for the extraction.

#### 2.1.2. Preparation of Dichloromethane: Methanol (DCM:M) Leaf Extracts

Different maturity stages (immature, partly mature, and mature) of powdered leaf samples of Ceylon cinnamon (2.5 g each) were separately extracted into 25 mL of dichloromethane:methanol in a ratio of (1:1, v/v) at room temperature for 7 days with occasional shaking. The extracts were separately filtered, evaporated under reduced pressure using a rotary evaporator, dried under nitrogen until constant weight obtained, and stored at −20°C until use for the analysis.

### 2.2. Chemicals and Reagents

Soluble starch, D-glucose, *α*-glucosidase (type V from rice), p-nitrophenyl *α*-D-glucopyranoside, acarbose, 3,5-dinitrosalicylic acid (DNS), Folin-Ciocalteu reagent, gallic acid, quercetin, 6-hydroxy-2-5-7-8-tetramethylchroman-2-carboxylic acid (Trolox), 1,1-diphenyl-2-picryl-hydrazyl (DPPH), 2,2-azino-bis(3-ethylbenzothiazoline-6-sulfonic acid) diammonium salt (ABTS), potassium persulphate, 2,2′-azobis (2-amidinopropane) dihydrochloride (AAPH), sodium fluorescein, 2,4,6-tripyridyl-s-triazine (TPTZ), and dimethyl sulfoxide (DMSO) were purchased from Sigma-Aldrich, USA. *α*-amylase (*Bacillus amyloliquefaciens*) was purchased from Roche Diagnostics, USA. All the other chemicals and reagents were of analytical grade. All the analyses were carried out using high-throughput 96-well microplate readers (SpectraMax Plus384, Molecular Devices, USA and SPECTRAmax-Gemini EM, Molecular Devices Inc, USA).

### 2.3. Methods

#### 2.3.1. Quantification of Antioxidants and Antioxidant Activity

*(1) Quantification of Antioxidants*

*(a) Total Polyphenolic Content (TPC). *The TPC of immature, partly mature, and mature leaf of Ceylon cinnamon was determined by the Folin-Ciocalteu method described by Singleton et al. [30] in 96-well microplates. Twenty microliters of 0.5 mg/mL of immature, partly mature and mature leaf extracts of Ceylon cinnamon were added to 110 *μ*L of ten times diluted freshly prepared Folin-Ciocalteu reagent. Seventy microliters of sodium carbonate solution was added to the mixture and incubated at room temperature (25 ± 2°C) for 30 minutes. The absorbance was recorded at 765 nm. Five different concentrations of 20 *μ*L of gallic acid (0.06, 0.12, 0.25, 0.5, and 1 mg/mL) were used to construct the standard curve. The results were expressed as mg gallic acid equivalents per 1 g of extract/1 g leaf sample of cinnamon.

*(b) Total Flavonoid Content (TFC)*. Total flavonoid content of immature, partly mature, and mature leaf extracts of Ceylon cinnamon was carried out by aluminium chloride method described by Siddhuraju and Becker [31] in 96-well microplates. One hundred microliters of 2% aluminium chloride in methanol solution was added to 100 *μ*L of 0.25 mg/mL of immature, partly mature, and mature leaf extracts of cinnamon in methanol. The mixture was incubated at room temperature (25 ± 2°C) for 10 minutes and absorbance was recorded at 415 nm. Preplate reading was recorded before addition of the aluminium chloride solution. Six different concentrations of 100 *μ*L of quercetin (7.81, 15.62, 31.25, 62.5, and 125 mg/mL) were used to construct the calibration curve. The results were expressed as mg quercetin equivalents per 1 g of extract/1 g of leaf sample of cinnamon. 

(*2) In Vitro Antioxidant Activity*

*(a) DPPH Radical Scavenging Activity. *The DPPH radical scavenging assay was performed according to the method described by Blois [32] in 96-well microplates. Reaction volumes of 200 *μ*L, containing 125 *μ*M of DPPH radical and 50 *μ*L of different concentrations of leaf extracts of cinnamon (immature leaf and partly mature leaf: 0.12, 0.25, 0.5, 1, 2 mg/mL; mature leaf: 15.62, 31.25, 62.5, 125, 250, 500 *μ*g/mL) were incubated at 25 ± 2°C for 15 minutes. The absorbance was recorded at 517 nm. Five different concentrations of 50 *μ*L of Trolox (3.125, 6.25, 12.5, 25, and 50 *μ*g/mL) were used to construct the standard curve. The results were expressed as mg Trolox equivalents per 1 g of extract/1 g of leaf sample of cinnamon.

*(b) ABTS Radical Scavenging Activity. *The ABTS^**+**^ radical scavenging assay was performed according to the method described by Re et al. [33] in 96-well microplates. A stable stock solution of ABTS radical cation was produced by reacting 10 mM of ABTS in potassium persulfate at 37°C for 16 hrs in dark. The reaction volume of 200 *μ*L containing 40 *μ*M of ABTS^+^ radical and 50 *μ*L of different concentrations of leaf extracts of cinnamon (immature leaf and partly mature leaf: 15.62, 31.25, 62.5, 125, 250 *μ*g/mL; mature leaf: 0.98, 1.95, 3.90, 7.81, 15.62, 31.25 *μ*g/mL) was incubated at 25 ± 2°C for 10 minutes. The absorbance was recorded at 734 nm. Five different concentrations of 50 *μ*L of Trolox (50, 25, 12.5, 6.25 and 3.12 *μ*g/mL) were used to construct the standard curve. The results were expressed as mg Trolox equivalents per 1 g of extract/1 g of leaf sample of cinnamon. 

*(c) Oxygen Radical Absorbance Capacity (ORAC)*. The ORAC radical scavenging assay was performed according to the method described by Ou et al. [34] with some modification in 96-well microplates. The assay was conducted at 37°C (pH 7.4) with a blank sample in parallel. Trolox standards (1.5 and 0.75 *μ*g/mL), fluorescein (4.8 *μ*M), and AAPH (40 mg/mL) solutions were prepared prior to use in phosphate buffer (75 mM, pH 7.4). Leaf samples were initially dissolved in dimethyl sulfoxide (DMSO) and concentration of DMSO in blank and samples were 0.125 *μ*L/mL. Reaction volume of 200 *μ*L, containing 100 *μ*L of 4.8 *μ*M fluorescein and 50 *μ*L of leaf extracts of cinnamon (immature leaf: 25 *μ*g/mL; partly mature leaf: 12.5 *μ*g/mL; mature leaf: 6.25 *μ*g/mL), was preincubated at 37°C for 10 minutes. Then, 50 *μ*L of AAPH (40 mg/mL) was added to each well to initiate the reaction. The plate was placed on the fluorescent microplate reader (SPECTRAmax-Gemini EM, Molecular Devices Inc., USA) set with excitation and emission at 494 nm and 535 nm and decay of fluorescein was recorded in 1 minute interval for 35 minutes. Trolox was used as a standard antioxidant. ORAC activities of the samples were calculated by comparing the net area under curve of fluorescein decay between the blank and the samples. The results were expressed as mg Trolox equivalents per 1 g of extract/1 g of leaf sample of cinnamon. 

*(d) Ferric Reducing Antioxidant Power (FRAP).* The assay was carried out according to the method of Benzie & Szeto [35] with some modifications in 96-well microplates. The working FRAP reagent was produced by mixing 300 mM acetate buffer (pH 3.6), 10 mM TPTZ solution, and 20 mM Iron (III) Chloride Hexahydrate (FeCl_3_.6H_2_O) in a ratio of 10:1:1 just before use and incubate at 37°C for 10 mins. The TPTZ solution was prepared by making a solution of 10 mM TPTZ in 40 mM HCl. Reaction volume of 200 *μ*L containing 150 *μ*L of working FRAP reagent, 30 *μ*L of acetate buffer, and 20 *μ*L of 250 *μ*g/mL of immature, partly mature, and mature leaf extracts of cinnamon was incubated at room temperature (30 ± 2°C) for 8 minutes. The absorbance was recorded at 600 nm. The results were expressed as mg of Trolox equivalents per 1 g of extract/1 g of leaf sample of cinnamon.

#### 2.3.2. Glycemic Regulatory Properties

*(1) Antiamylase Activity*. The antiamylase activity of different maturity stages of DCM:M leaf extracts of Ceylon cinnamon was carried out according to the method of Bernfeld [36] with some modifications. Briefly, a reaction volume of 1 mL containing 50 *μ*L of DCM:M leaf extracts (immature, partly mature, and mature leaf extracts: 312.5, 625, 1250, and 2500 *μ*g/mL, n = 3 each), 40 *μ*L of starch (1% w/v), and 50 *μ*L of enzyme (5 *μ*g/mL) in 100 mM sodium acetate buffer (pH 6.0) was incubated at 40°C for 15 min. Then, 0.5 mL of DNS reagent was added, placed in a boiling water bath for 5 min, and allowed to cool in an ice water bath. The absorbance was recorded at 540 nm using 96-well microplate reader (SPECTRAmaxPLUS384 Molecular Devices, Inc., USA). Control experiments were conducted in an identical way replacing extracts with 50 *μ*L of 100 mM sodium acetate buffer. For sample blanks the enzyme solutions were replaced with acetate buffer and the same procedure was carried out. Acarbose was used as the positive control (6.25-100 *μ*g/mL, n = 4). Antiamylase activity was given as (% inhibition). Inhibition % was calculated using following equation:

(1) $$\begin{array}{c} Inhibition\;\left(\;\%\;\right)=\left[\;A_{c}-\frac{\left(A_{s}-A_{b}\right)}{A_{c}}\;\right]*100 \end{array}$$

where A_c_ is the absorbance of the control, A_b_ is the absorbance of sample blanks, and A_s_ is the absorbance in the presence of leaf extracts or acarbose.

(*2) Antiglucosidase Activity. *Antiglucosidase activity of different maturity stages of DCM:M leaf extracts of Ceylon cinnamon was carried out according to the method of Matsui et al. [37] with slight modification in 96-well microplates. A reaction volume of 0.1 mL containing 4 mM p-nitrophenyl-*α*-D-glucopyranoside, 50 mU/mL of *α*-glucosidase, and 40 *μ*L of leaf extracts (immature, partly mature, and mature leaf: 78.12, 156.25, 312.50, 625, 1250 *μ*g/mL; n=3) in 50 mM sodium acetate buffer (pH 5.8) was incubated at 37°C for 30 min. The reaction was then stopped by addition of 50 *μ*L of 0.1 M Na_2_CO_3_ and the absorbance was recorded at 405 nm using 96-well microplate reader. Reaction mixture without extract was used as the control and reaction mixture with the extract and without enzyme was used as the sample blank. Acarbose, a clinical *α*-glucosidase inhibitor, was used as the positive control (0.25–2.50 *μ*g/mL, n = 4). Antiglucosidase activity (% inhibition) was calculated by using following equation:

(2) $$\begin{array}{c} Inhibition\;\left(\;\%\;\right)=\left[\;A_{c}-\frac{\left(A_{s}-A_{b}\right)}{A_{c}}\;\right]*100 \end{array}$$

where A_c_ is the absorbance of the control (100% enzyme activity), A_b_ is the absorbance produced by cinnamon extracts (sample blank), and A_s_ is the absorbance of the sample in the presence of cinnamon leaf extracts or acarbose.

#### 2.3.3. Statistical Analysis

Data of each experiment were statistically analyzed using SAS software version 6.12. One-way analysis of variance (ANOVA) and the Duncan's Multiple Range Test (DMRT) were used to determine the differences among treatment means. Pearson's correlation coefficient was used for the correlation analysis. P < 0.05 was regarded as significant.

## 3. Results

### 3.1. Percentage Extractables of Different Maturity Stages (Immature, Partly Mature, and Mature) of Leaf of Ceylon cinnamon

Percentage extractables of different maturity stages (immature, partly mature, and mature) of leaf of Ceylon cinnamon are given in Table 1. Percentage extractables of immature, partly mature, and mature leaves of Ceylon cinnamon were ranged from 2.53 to 10.39%. Mature leaf had the highest extractables (10.39%) whereas immature leaf had the lowest extractables (2.53%). The order of % extractables of different maturity stages of Ceylon cinnamon were mature > partly mature > immature.

### 3.2. Antioxidants and Antioxidant Activity

#### 3.2.1. Antioxidants (Total Polyphenolic and Total Flavonoid Contents) of Different Maturity Stages of Leaf of Ceylon Cinnamon

Total polyphenolic content and total flavonoid content of immature, partly mature, and mature leaf of Ceylon cinnamon were given in Table 2. The mean TPC and TFC of immature, partly mature, and mature leaves of Ceylon cinnamon were ranged from 26.80 ± 0.77 to 215.07 ± 1.98 mg gallic acid equivalents per g of extract/0.68 ± 0.02 to 22.35 ± 0.21 mg gallic acid equivalents per g of sample and 33.65 ± 0.30 to 45.01 ± 0.56 mg quercetin equivalents per g of extract/0.85 ± 0.01 to 4.68 ± 0.06 mg quercetin equivalents per g of sample, respectively. Significant differences were observed for both TPC and TFC among different maturity stages of leaf of Ceylon cinnamon (p < 0.05). Further, mature leaf of Ceylon cinnamon had the highest TPC (215.07 ± 1.98 mg gallic acid equivalents per g of extract/22.35 ± 0.21 mg gallic acid equivalents per g of sample) and TFC (45.01 ± 0.56 mg quercetin equivalents per g of extract/4.68 ± 0.06 mg quercetin equivalents per g of sample). In contrast immature leaf extract had the lowest TPC (26.80 ± 0.77 mg gallic acid equivalents per g of extract/0.68 ± 0.02 mg gallic acid equivalents per g of sample) and TFC (33.65 ± 0.30 mg quercetin equivalents per g of extract/0.85 ± 0.01 mg quercetin equivalents per g of sample). The order of mean TPC and TFC contents in different maturity stages of leaf of Ceylon cinnamon were mature > partly mature > immature.

#### 3.2.2. Antioxidant Activity of Different Maturity Stages of Leaf of Ceylon cinnamon

DPPH, ABTS and ORAC radical scavenging activities and ferric reducing antioxidant power of different maturity stages of leaf of Ceylon cinnamon is given in Table 3. All maturity stages including immature, partly mature and mature leaf of Ceylon cinnamon demonstrated antioxidant activity in terms of radical scavenging by DPPH, ABTS and ORAC and reducing power by FRAP method. However, results clearly revealed significant differences (p < 0.05) among different maturity stages of leaf of Ceylon cinnamon for antioxidant activity. Mature leaf of Ceylon cinnamon had the highest antioxidant activity for all the studied antioxidant assays (DPPH: 260.66 ± 6.21 mg Trolox equivalents per g of extract/27.09 ± 0.65 mg Trolox equivalents per g of sample; ABTS: 422.46 ± 14.03 mg Trolox equivalents per g of extract/43.91 ± 1.46 mg Trolox equivalents per g of sample; ORAC: 179.97 ± 2.51 mg Trolox equivalents per g of extract/18.70 ± 0.26 mg Trolox equivalents per g of sample; FRAP: 665.44 ± 5.05 mg Trolox equivalents per g of extract/69.16 ± 0.52 mg Trolox equivalents per g of sample). Whereas immature leaf of Ceylon cinnamon had the lowest antioxidant activity (DPPH: 260.66 ± 6.21 mg Trolox equivalents per g of extract/27.09 ± 0.65 mg Trolox equivalents per g of sample; ABTS: 422.46 ± 14.03 mg Trolox equivalents per g of extract/43.91 ± 1.46 mg Trolox equivalents per g of sample; ORAC: 179.97 ± 2.51 mg Trolox equivalents per g of extract/18.70 ± 0.26 mg Trolox equivalents per g of sample; FRAP: 665.44 ± 5.05 mg Trolox equivalents per g of extract/69.16 ± 0.52 mg Trolox equivalents per g of sample). Further, the order of potency for antioxidant activity was mature leaf > partly mature leaf > immature leaf. Moreover, the dose response relationship of different maturity stages (immature, partly mature, and mature) of leaf of Ceylon cinnamon for DPPH and ABTS radical scavenging activities is given in Tables 4 and 5 respectively.

### 3.3. Glycemic Regulatory Properties

#### 3.3.1. Antiglucosidase Activity

Antiglucosidase activity of different maturity stages of leaf of Ceylon cinnamon is given in Table 6. Immature, partly mature and mature leaf extracts of Ceylon cinnamon did not demonstrate antiglucosidase activity at the studied concentration of 400 *μ*g/mL.

#### 3.3.2. Antiamylase Activity

Antiamylase activity of different maturity stages of leaf of Ceylon cinnamon is given in Table 7. Immature, partly mature, and mature leaf of Ceylon cinnamon showed antiamylase activity with varying potential. The % inhibition of amylase enzyme by immature, partly mature, and mature leaf of Ceylon cinnamon were ranged from 5.80 ± 1.67 to 18.05 ± 0.24, 10.22 ± 2.24 to 27.81 ± 1.09, and 4.97 ± 1.32 to 36.62 ± 4.00, respectively. Mature leaf had the highest inhibition of *α*-amylase enzyme whereas immature leaf showed the lowest inhibition at 2.5 mg/mL concentration. However, compared to the standard drug acarbose all maturity stages of leaf of Ceylon cinnamon showed moderate antiamylase activity. Further, the order of potency of leaf extracts of Ceylon cinnamon for antiamylase activity were mature > partly mature > immature.

## 4. Discussion

Antioxidant and glycemic regulatory properties of different maturity stages of authenticated leaves of Ceylon cinnamon were evaluated using well established, widely used, sensitive, specific, validated, and internationally accepted antioxidant and antidiabetic related bioassays* in vitro* [31–37]. Leaf extracts were evaluated for antidiabetic related properties since leaf of Ceylon cinnamon is claimed to have antidiabetic activity in Sri Lankan traditional knowledge [26] and folklore. DCM:M extracts of different maturity stages of leaf of Ceylon cinnamon were studied since DCM:M leaf extract of Ceylon cinnamon was previously studied for antioxidant [29] and antidiabetic activities* in vitro* [28].

Polyphenols in spices are secondary metabolites and are a large family of structurally diverse compounds. Numerous studies have shown that long-term consumption of spice polyphenolics offers protection against multiple degenerative diseases [38]. According to the United States Department of Agriculture (USDA), cinnamon is one of the most important spices consume by people world over [39] and reported to be a rich source of polyphenolics [7, 40–42]. Although bark of cinnamon is widely investigated for phenolic composition, leaf extract is not thoroughly studied for its phenolic composition to date. A study carried out by Prasad et al. [43] reported that 50% ethanolic leaf extracts of various* Cinnamomum *species such as* C. zeylanicum*,* C. cassia*,* C. burmannii, C. tamala, *and* C. pauciflorum *had total polyphenolic content as 2708.7 ± 60.6, 1558.7 ± 46.4, 943.7 ± 29.29, 694.4 ± 32.3, and 1337.2 ± 26.2 *μ*g gallic acid equivalents/g of sample, respectively, and highlighted that leaf of* C. zeylanicum *had the highest total polyphenolic content. We have recently reported the total polyphenolic content of leaf of Ceylon cinnamon (192.83 ± 6.31 to 266.28 ± 9.97 mg gallic acid equivalents/g of extract or 20.18 ± 0.70 and 44.57 ± 1.70 mg gallic acid equivalents/g of cinnamon) [29], and it was significantly higher than the values reported by Prasad et al. [43]. However, to date total polyphenolic content of different maturity stages of leaf of Ceylon cinnamon is not reported. Therefore, this is the first report on total phenolic content of different maturity stages of leaf of Ceylon cinnamon and results highlighted that immature and partly mature leaf possess nearly 30 and 10 times lower TPC, respectively, than its mature leaf.

Flavonoids are the predominant class among polyphenolics in plants and recent interest on these substances has been stimulated by their potential health benefits through numerous* in vitro*,* in vivo,* and recent clinical studies [44]. The total flavonoid content of different maturity stages of Ceylon cinnamon varies from 0.85 ± 0.01 to 4.68 ± 0.06 mg quercetin equivalents/g of sample. The mature leaf had the highest total flavonoid content; however, the % TFC/TPC ratio was significantly decreased with leaf maturity (% TFC/TPC ratio: immature, partly mature, and mature leaf: 125, 80, and 21%, respectively). Although there were previous reports on TFC of leaf extracts of different* Cinnamomum* species including* C. zeylanicum* (*C. cassia*: 981.1 ± 66.6;* C. zeylanicum*: 1075 ± 13.8;* C. burmannii*: 2738.4 ± 10.2;* C. tamala*: 568.1 ± 9.7;* C. pauciflorum*: 1564.4 ± 14 *μ*g quercetin equivalents/g of sample [43];* C. cassia*: 33.48 ± 2.90 mg quercetin equivalents/g of extract [45];* C. zeylanicum*: 6.80 ± 0.12-12.00 ± 0.37 mg quercetin equivalents/g of leaf [29]) this is the first report on TFC content of different maturity stages of leaf of Ceylon cinnamon.

The antioxidant activity of different maturity stages of leaf extracts of Ceylon cinnamon was investigated via several* in vitro *antioxidant bioassay methods which included radical scavenging activity by DPPH, ABTS, and ORAC and reducing power by FRAP.

Investigated all maturity stages of leaf extracts of Ceylon cinnamon demonstrated radical scavenging activity by all the studied radical scavenging mechanisms such as DPPH, ABTS, and ORAC methods. Further, all maturity stages of leaf of Ceylon cinnamon showed significantly (p < 0.05) high ABTS radical scavenging activity than DPPH and ORAC radical scavenging mechanisms. The order of potency of radical scavenging activities of different maturity stages of Ceylon cinnamon was ABTS > ORAC (except mature leaf) > DPPH. Previous reports too showed that leaf extracts of* Cinnamomum cassia* and* Cinnamomum zeylanicum* (*C. verum*) possess radical scavenging activities by DPPH and ABTS mechanisms [29, 42, 43, 45] and according to their reports leaf extract of* C. zeylanicum* (*C. verum*) had the highest DPPH and ABTS radical scavenging activities. We have previously reported the ORAC radical scavenging activity of leaf extract of Ceylon cinnamon; interestingly it was the first report on ORAC of leaf of any* Cinnamomum *species worldwide [29]. However, radical scavenging activities of different maturity stages of leaf extracts of Ceylon cinnamon are not reported to date since this is the first report.

Reducing power of bioactive compounds is associated with antioxidant activity. In general reducing properties are associated with the presence of reductones [46]. It is reported that the antioxidant action of reductones is based on the breaking of the free radical chain by donating a hydrogen atom, or reacting with certain precursors of peroxide to prevent peroxide formation [46]. Further, Shimada et al. [47] reported that reducing power of cinnamon leaf might be due to the di and monohydroxyl substitutions in the aromatic ring, which possess potent hydrogen donating abilities. Previous studies on FRAP of leaf extracts of different* Cinnamomum *species indicated that leaf of Ceylon cinnamon possess the highest FRAP value (absorbance at 700 nm for 50 *μ*g/mL ~ 1.1[43]; 65.17 ± 2.33 to 125.71 ± 3.21 mg FeSO4/g leaf on dry weight basis or absorbance at 700 nm for 50 *μ*g/mL ~ 1.05-1.30 [29]) than other* Cinnamomum *species world over. However, this is the first report on ferric reducing antioxidant power of different maturity stages of leaf of Ceylon cinnamon and results highlighted that mature leaf had nearly 55 and 39 times higher FRAP value compared to the immature and partly mature leaves of Ceylon cinnamon.

*α*-Amylase and *α*-glucosidases are the key enzymes involved in starch digestion process [7]. Thus, inhibitors of these enzymes can play a key role in the management of diabetes. Compared to the bark, leaf of* Cinnamomum *species was rarely investigated for antiamylase activity to date. A research carried out by Ponnusamy et al. [48] reported antiamylase activity of leaf extract of* C. verum *as IC_50_ value 1 *μ*g/mL. Further, Arachchige et al. [28] reported that ethanolic and DCM:M leaf extracts of Ceylon cinnamon possess antiamylase activity and activity as IC_50_ 943 ± 28 *μ*g/mL and 17.59 ± 1.24% inhibition at 1.5 mg/mL, respectively. To the best of my knowledge these two reports are the only available reports on antiamylase activity of leaf of* Cinnamomum *species worldwide. However, to date different maturity stages of leaf extracts of none of the* Cinnamomum* species are reported to have antiamylase activity. Therefore, this is the first report on antiamylase activity of leaf extracts of different maturity stages of leaf of any* Cinnamomum* species world over and results highlighted that mature leaf had the highest antiamylase activity (36.62 ± 4.00% inhibition) and immature leaf had the lowest activity (18.05 ± 0.24% inhibition) at 2.5 mg/mL assay concentration. Further, none of the investigated different maturity stages of leaf extracts of Ceylon cinnamon showed antiglucosidase activity.

Several research findings have clearly shown that antioxidants and phenolic compounds have significant positive correlation with antiamylase and other antidiabetic related activities such as acetylcholine and butyrylcholine esterases inhibitory activities [8, 10, 49, 50]. Further, proanthocyanidin, a phytochemical class among phenolics, is reported shown antidiabetic activity in cinnamon via different mechanisms [9, 11]. In our previous study it is reported that ethanolic and DCM:M leaf extracts of Ceylon cinnamon had total phenolics and total proanthocyanidins in varying quantities. Therefore, observed antiamylase activity of different maturity stages of leaf of Ceylon cinnamon may be, at least partly, due to the phenolics particularly as proanthocyanidins. The observed differences in antiamylase activity among different maturity stages of leaves of Ceylon cinnamon may be ascribed to the differences in composition and concentration of bioactive compounds (phenolics particularly as proanthocyanidins) in each maturity stage of leaf of Ceylon cinnamon [29, 48].

Ceylon cinnamon leaf is used in traditional medicine however; the maturity stage needed to be taken is not properly mentioned. Hence the present paper highlighted the importance of selecting correct maturity stage when use in traditional medicine. Further, commercial importance of Ceylon cinnamon leaf is to extract leaf essential oil. Leaf essential oil is a mixture of compounds with many health benefits and it has many applications. When extracting leaf essential oil the common practice is to use all maturity stages of cinnamon leaf. However, the composition of leaf essential oil is different with different batches. The one of the reasons may be due to the use of combinations of different maturity stages of leaf in each batch. So present study may also pave a path way to investigate that issue which is an urgent requirement in trade. Further, the present study scientifically validated for the first time on antioxidant and glycemic regulatory properties of different maturity stages of leaf of Ceylon cinnamon. Moreover, in summary results clearly demonstrated the importance of selecting the right maturity stage for the development of value added functional products and use in traditional medicinal formulations and also for the extraction of leaf essential oil since currently Sri Lanka is the only continuous supplier of cinnamon leaf essential to the world market.

## 5. Conclusions

It is concluded that all the maturity stages of leaf of Ceylon cinnamon possess antioxidant and glycemic regulatory properties with varying degrees of potential. Mature leaf demonstrated the highest antioxidant and glycemic regulatory properties and immature leaf showed the lowest. The order of potency of leaf extracts of Ceylon cinnamon for antioxidant and glycemic regulatory properties were mature > partially mature > immature. Therefore, findings of the present study clearly highlighted the use of correct maturity stage when use in traditional medicine and in extraction of leaf essential oil. Further, this is the first study to report antioxidant and glycemic regulatory properties of different maturity stages of leaf of Ceylon cinnamon and highlights its potential in managing of oxidative stress-associated chronic diseases.

## Figures and Tables

**Table 1:** Percentage extractables of different maturity stages of leaf of Ceylon cinnamon.

Leaf extract	% Extractables
Immature	2.53
Partly mature	5.38
Mature	10.39

**Table 2:** Total polyphenolic content and total flavonoid content of different maturity stages of leaf of Ceylon cinnamon.

Leaf extract	Antioxidants			
	TPC		TFC	
	(mg gallic acid equivalents/g of extract)	(mg gallic acid equivalents/g of sample)	(mg quercetin equivalents/g of extract)	(mg quercetin equivalents/g of sample)
Immature	26.80 ± 0.77^c^	0.68 ± 0.02^c^	33.65 ± 0.30^c^	0.85 ± 0.01^c^
Partly mature	44.82 ± 0.37^b^	2.41 ± 0.02^b^	35.78 ± 0.52^b^	1.93 ± 0.03^b^
Mature	215.07 ± 1.98^a^	22.35 ± 0.21^a^	45.01 ± 0.56^a^	4.68 ± 0.06^a^

Data represented as mean ± SEM. TPC and TFC n=4 each. Values in a column superscripted by different letters are significantly different at p < 0.05.

**Table 3:** Antioxidant activity of different maturity stages of leaves of Ceylon cinnamon.

Leaf extract	Antioxidant activity							
	DPPH		ABTS		ORAC		FRAP	
	(mg Trolox equivalents/g of extract)	(mg Trolox equivalents/g of sample)	(mg Trolox equivalents/g of extract)	(mg Trolox equivalents/g of sample)	(mg Trolox equivalents/g of extract)	(mg Trolox equivalents/g of sample)	(mg Trolox equivalents/g of extract)	(mg Trolox equivalents/g of sample)
Immature	16.54 ± 0.32^c^	0.42 ± 0.01^c^	140.67 ± 4.00^c^	3.57 ± 0.10^c^	27.86 ± 0.55^c^	0.71 ± 0.01^c^	12.11 ± 0.98^c^	0.31 ± 0.02^c^
Partly mature	18.32 ± 0.23^b^	0.99 ± 0.01^b^	227.63 ± 20.45^b^	12.25 ± 1.10^b^	46.72 ± 3.72^b^	2.51 ± 0.20^b^	17.01 ± 1.40^b^	0.92 ± 0.08^b^
Mature	260.66 ± 6.21^a^	27.09 ± 0.65^a^	422.46 ± 14.03^a^	43.91 ± 1.46^a^	179.97 ± 2.51^a^	18.70 ± 0.26^a^	665.44 ± 5.05^a^	69.16 ± 0.52^a^

Data represented as mean ± SEM. DPPH, ABTS, and ORAC n=4 each; FRAP n=3 each. Values in a column superscripted by different letters are significantly different at p < 0.05.

**Table 4:** Dose response relationship of different maturity stages of leaf of Ceylon cinnamon for DPPH radical scavenging activity.

Leaf extract	(% Inhibition)					
	Concentration (*μ*g/mL)					
	125	250	500	1000	2000	IC_50_ (*μ*g/mL)
Immature	15.82 ± 0.46	26.22 ± 0.27	47.31 ± 0.57	61.66 ± 0.51	72.98 ± 0.56	*525.18 ± 10.04* ^*a*^
Partly mature	16.08 ± 0.58	30.26 ± 0.14	52.57 ± 0.27	67.76 ± 0.31	82.15 ± 0.57	*473.95 ± 6.16* ^*b*^
	*15.62*	*31.25*	*62.50*	*125*	*250*	*IC*_*50*_
Mature	29.21 ± 1.57	55.98 ± 0.46	74.41 ± 0.14	86.36 ± 0.27	91.25 ± 0.26	*33.35 ± 0.78* ^*c*^

Data represented as mean ± SEM (n = 4 for each leaf extracts and n = 3 for Trolox); values in the column superscripted by different letters are significantly different at p < 0.05; immature, partly mature, mature leaf extracts, and Trolox r^2^: 1.00, 1.00, 0.92, and 1.00, respectively; IC_50_: Trolox 8.68 ± 0.05 *μ*g/mL.

**Table 5:** Dose response relationship of different maturity stages of leaf of Ceylon cinnamon for ABTS radical scavenging activity.

Leaf extract	(% Inhibition)					
	Concentration (*μ*g/mL)					
	15.62	31.25	62.50	125	250	IC_50_
Immature	28.50 ± 2.17	67.39 ± 4.18	87.59 ± 2.29	96.25 ± 0.23	96.28 ± 0.39	*45.28 ± 1.29* ^*a*^
Partly mature	22.13 ± 2.54	59.42 ± 7.40	83.55 ± 5.47	94.81 ± 2.02	98.16 ± 0.71	*28.51 ± 2.87* ^*b*^
	*1.95*	*3.90*	*7.81*	*15.62*	*31.25*	*IC*_*50*_
Mature	5.59 ± 4.69	16.27 ± 2.10	46.32 ± 2.25	54.58 ± 3.56	96.43 ± 1.73	*15.09 ± 0.51* ^*c*^

Data represented as mean ± SEM (n = 4 each for leaf extracts and n = 3 for Trolox); values in the column superscripted by different letters are significantly different at p < 0.05; immature, partly mature, mature leaf extracts, and Trolox r^2^: 0.92, 0.94, 0.94, and 1.00, respectively; IC_50_: Trolox 6.36 ± 0.03 *μ*g/mL.

**Table 6:** Antiglucosidase activity of different maturity stages of leaf of Ceylon cinnamon.

Leaf extract	% Inhibition
Immature	-6.24 ± 1.14
Partly mature	-4.57 ± 0.48
Mature	-9.92 ± 5.11

Data represented as mean ± SEM (n=3 each). % inhibition at 1250 *μ*g/mL; IC_50_ acarbose 0.47 ± 0.01 *μ*g/mL.

**Table 7:** Antiamylase activity of different maturity stages (immature, partly mature, and mature) of leaf of Ceylon cinnamon.

Leaf extract	% Inhibition			
	Concentration (*μ*g/mL)			
	312.5	625	1250	2500
Immature	5.80 ± 1.67	11.89 ± 1.88	15.37 ± 0.82	18.05 ± 0.24
Partly mature	10.22 ± 2.24	11.35 ± 1.76	22.17 ± 2.04	27.81 ± 1.09
Mature	4.97 ± 1.32	11.09 ± 0.49	17.73 ± 4.53	36.62 ± 4.00

Data represented as mean ± SEM (n = 3). Immature leaf, partly mature leaf, and mature leaf r^2^ = 0.96, 0.91, and 0.99, respectively; IC_50_ acarbose: 113.88 ± 2.54 *μ*g/mL; DCM:M dichloromethane:methanol.

## Data Availability

The data used to support the findings of this study are available from the corresponding author upon request.

## References

[1] Lobo V., Patil A., Phatak A., Chandra N. (2010). Free radicals, antioxidants and functional foods: impact on human health. *Pharmacognosy Reviews*.

[2] Birben E., Sahiner U. M., Sackesen C., Erzurum S., Kalayci O. (2012). Oxidative stress and antioxidant defense. *World Allergy Organization Journal*.

[3] International Diabetes Federation (2014). *Diabetes Atlas, International Diabetes Federation, Brussels*.

[4] American Diabetes Association (2015). Classification and diagnosis of diabetes. *Diabetes Care*.

[5] International Diabetes Federation (2017). *Diabetes Atlas*.

[6] Chaudhury A., Duvoor C., Reddy Dendi V. S. (2017). Clinical review of antidiabetic drugs: Implications for type 2 diabetes mellitus management. *Frontiers in Endocrinology*.

[7] Adisakwattana S., Lerdsuwankij O., Poputtachai U., Minipun A., Suparpprom C. (2011). Inhibitory activity of cinnamon bark species and their combination effect with acarbose against intestinal *α*-glucosidase and pancreatic *α*-amylase. *Plant Foods for Human Nutrition*.

[8] Premakumara G. A. S., Abeysekera W. K. S. M., Ratnasooriya W. D., Chandrasekharan N. V., Bentota A. P. (2013). Antioxidant, anti-amylase and anti-glycation potential of brans of some Sri Lankan traditional and improved rice (*Oryza sativa* L.) varieties. *Journal of Cereal Science*.

[9] Peng X., Ma J., Chao J. (2010). Beneficial Effects of Cinnamon Proanthocyanidins on the Formation of Specific Advanced Glycation Endproducts and Methylglyoxal-Induced Impairment on Glucose Consumption. *Journal of Agricultural and Food Chemistry*.

[10] Khan M. T. H., Orhan I., Şenol F. S. (2009). Cholinesterase inhibitory activities of some flavonoid derivatives and chosen xanthone and their molecular docking studies. *Chemico-Biological Interactions*.

[11] Peng X., Cheng K.-W., Ma J. (2008). Cinnamon bark proanthocyanidins as reactive carbonyl scavengers to prevent the formation of advanced glycation endproducts. *Journal of Agricultural and Food Chemistry*.

[12] Nowotny K., Jung T., Höhn A., Weber D., Grune T. (2015). Advanced glycation end products and oxidative stress in type 2 diabetes mellitus. *Biomolecules*.

[13] Tan D., Wang Y., Lo C.-Y., Ho C.-T. (2008). Methylglyoxal: Its presence and potential scavengers. *Asia Pacific Journal of Clinical Nutrition*.

[14] Reddy V. P., Beyaz A. (2006). Inhibitors of the Maillard reaction and AGE breakers as therapeutics for multiple diseases. *Drug Discovery Therapy*.

[15] Ahmed N. (2005). Advanced glycation endproducts—role in pathology of diabetic complications. *Diabetes Research and Clinical Practice*.

[16] DeGroot J. (2004). The AGE of the matrix: chemistry, consequence and cure. *Current Opinion in Pharmacology*.

[17] Zatalia S. R., Sanusi H. (2013). The role of antioxidants in the pathophysiology, complications, and management of diabetes mellitus. *Acta medica Indonesiana*.

[18] Ravindran P. N., Babu K. N., Shylaja M. (2004). *CRC PRESS LLC*.

[19] Wijesekera R. O. B., Ponnuchamy S., Jayewardene A. L. (1975). *Ceylon Institute of Scientific and Industrial Research (CISIR)*.

[20] Abeysekera W. P. K. M. (2018). *Assessment of Some Potential Health Benefits of Sri Lankan Cinnamon, Cinnamomum Zeylanicum Blume (Cinnamomum Verum Presl) by Studying Selected Bioactivities [Ph.D. thesis]*.

[21] FAOSTAT http://www.fao.org/faostat/en/#home.

[22] Abeysekera W. P. K. M., Arachchige S. P. G., Ratnasooriya W. D. (2017). Bark extracts of ceylon cinnamon possess antilipidemic activities and bind bile acids in vitro. *Evidence-Based Complementary and Alternative Medicine*.

[23] Medagama A. B. (2015). The glycaemic outcomes of Cinnamon, a review of the experimental evidence and clinical trials. *Nutrition Journal *.

[24] Anonymous, Talpatha Osu Mahima: Extracted from ancient Sri Lankan palm leaf manuscripts/Ola leaf writings. Volume I, II, III, Department of Ayurveda, Bandaranayake Memorial Ayurvedic Research Institute: Deepani Publishers Pvt Ltd, Sri Lanka, 2002

[25] Jayaweera D. M. A. (1982). *Medicinal Plants (Indigenous And Exotic) Used in Ceylon*.

[26] Anonymous

[27] Chi C. Y. (1961). *New Chinese Materia Medica*.

[28] Arachchige S. P. G., Abeysekera W. P. K. M., Ratnasooriya W. D. (2017). Anti-amylase, anti-cholinesterases, anti-glycation and glycation reversing potential of bark and leaf of ceylon cinnamon (cinnamomum zeylanicum blume) in vitro. *Evidence Based Complementary and Alternative Medicine*.

[29] Abeysekera W. P. K. M., Premakumara G. A. S., Ratnasooriya W. D. (2013). In vitro antioxidant properties of bark and leaf extracts of ceylon cinnamon (cinnamomum zeylanicum blume. *Tropical Agricultural Research*.

[30] Singleton V. L., Orthofer R., Lamuela-Raventós R. M. (1999). Analysis of total phenols and other oxidation substrates and antioxidants by means of folin-ciocalteu reagent. *Methods in Enzymology*.

[31] Siddhuraju P., Becker K. (2003). Antioxidant properties of various solvent extracts of total phenolic constituents from three different agro climatic origins of drumstick tree (Moringa oleifera Lam.) leaves. *Journal of Agricultural and Food Chemistry*.

[32] Blois M. S. (1958). Antioxidant determinations by the use of a stable free radical. *Nature*.

[33] Re R., Pellegrini N., Proteggente A., Pannala A., Yang M., Rice-Evans C. (1999). Antioxidant activity applying an improved ABTS radical cation decolorization assay. *Free Radical Biology & Medicine*.

[34] Ou B., Hampsch-Woodill M., Prior R. L. (2001). Development and validation of an improved oxygen radical absorbance capacity assay using fluorescein as the fluorescent probe. *Journal of Agricultural and Food Chemistry*.

[35] Benzie I. F. F., Szeto Y. T. (1999). Total antioxidant capacity of teas by the ferric reducing/antioxidant power assay. *Journal of Agricultural and Food Chemistry*.

[36] Bernfeld P. (1955). Amylases, alpha and beta. *Methods in Enzymology*.

[37] Matsui T., Ueda T., Oki T., Sugita K., Terahara N., Matsumoto K. J. (2001). Alpha-glucosidas inhibition isolated anthocyanins. *Journal of Agricultural and Food Chemistry*.

[38] Opara E., Chohan M. (2014). Culinary herbs and spices: their bioactive properties, the contribution of polyphenols and the challenges in deducing their true health benefits. *International Journal of Molecular Sciences*.

[39] Hariri M., Ghiasvand R. (2016). Cinnamon and chronic diseases. *Drug Discovery from Mother Nature, Advances in Experimental Medicine and Biology*.

[40] Akowuah G. A., Ahmad M., Tan S. C., Yam M. F. (2013). GC-MS determination of major bioactive constituents and anti-oxidative activities of aqueous extracts of cinnamomum burmannii blume stem. *Natural Products Journal*.

[41] Dudonne S., Vitrac X., Coutiére P., Woillez M., Mérillon J.-M. (2009). Comparative study of antioxidant properties and total phenolic content of 30 plant extracts of industrial interest using DPPH, ABTS, FRAP, SOD and ORAC assays. *Journal of Agricultural and Food Chemistry*.

[42] Mathew S., Abraham T. E. (2006). *In vitro* antioxidant activity and scavenging effects of *Cinnamomum verum* leaf extract assayed by different methodologies. *Food and Chemical Toxicology*.

[43] Prasad K. N., Yang B., Dong X. (2009). Flavonoid contents and antioxidant activities from cinnamomum species. *Innovative Food Science and Emerging Technologies*.

[44] Shahidi F., Ambigaipalan P. (2015). Phenolics and polyphenolics in foods, beverages and spices: Antioxidant activity and health effects - A review. *Journal of Functional Foods*.

[45] Yang C., Li R., Chuang L. (2012). Antioxidant activity of various parts of cinnamomum cassia extracted with different extraction methods. *Molecules*.

[46] Duh P. D. (1998). Antioxidant activity of burdock (Arctium lappa linné): Its scavenging effect on free-radical and active oxygen. *Journal of the American Oil Chemists' Society*.

[47] Shimada K., Fujikawa K., Yahara K., Nakamura T. (1992). Antioxidative properties of xanthan on the autoxidation of soybean oil in cyclodextrin emulsion. *Journal of Agricultural and Food Chemistry*.

[48] Kumar A. R., Ponnusamy S., Ravindran R., Zinjarde S., Bhargava S. (2011). Evaluation of traditional indian antidiabetic medicinal plants for human pancreatic amylase inhibitory effect in vitro. *Evidence-Based Complementary and Alternative Medicine*.

[49] Duru M. E., Tel G., Öztürk M., Harmandar M. (2012). Chemical composition, antioxidant and anticholinesterase activities of the essential oil of salvia chrysophylla staph. *Records of Natural Products*.

[50] Topcu G., Kolak U., Ozturk M. (2013). Investigation of anticholinesterase activity of a series of salvia extracts and the constituents of salvia staminea. *The Natural Products Journal*.

